# Characterization of Inhibitory GABA-A Receptor Activation during Spreading Depolarization in Brain Slice

**DOI:** 10.1371/journal.pone.0110849

**Published:** 2014-10-22

**Authors:** Isamu Aiba, C. William Shuttleworth

**Affiliations:** Department of Neurosciences, University of New Mexico School of Medicine, Albuquerque, New Mexico, United States of America; Dalhousie University, Canada

## Abstract

Spreading depolarization (SD) is a slowly propagating wave of near complete depolarizations of neurons and glia. Previous studies have reported large GABA releases during SD, but there is limited understanding of how GABA release and receptor activation are regulated and influence the propagating SD wavefront, as well as an excitatory phase immediately following the passage of SD. The present study characterized GABA-A type receptor (GABA_A_R) currents during SD generated by KCl microinjection in acute hippocampal slices from adult mice. Spontaneous GABA_A_R-mediated currents (sIPSCs) were initially enhanced, and were followed by a large outward current at the wavefront. sIPSC were then transiently supressed during the late SD phase, resulting in a significant reduction of the sIPSC/sEPSC ratio. The large outward current generated during SD was eliminated by the GABA_A_R antagonist gabazine, but the channel potentiator/agonist propofol failed to potentiate the current, likely because of a ceiling effect. Extracellular Cl^−^ decreases recorded during SD were reduced by the antagonist but were not increased by the potentiator. Together with effects of GABA_A_R modulators on SD propagation rate, these results demonstrate a significant inhibitory role of the initial GABA_A_R activation and suggest that intracellular Cl^−^ loading is insufficient to generate excitatory GABA_A_R responses during SD propagation. These results provide a mechanistic explanation for facilitating effects of GABA_A_R antagonists, and the lack of inhibitory effect of GABA_A_R potentiators on SD propagation. In addition, selective suppression of GABA transmission in the late SD period and the lack of effect of GABA_A_ modulators on the duration of SD suggests that GABA modulation may not be effective approach to protect neurons during the vulnerable phase of SD.

## Introduction

Recent evidence has suggested that the phenomenon of spreading depolarization (SD) can be a significant contributor to the progression of acute brain injury, and also for other pathophysiological events such as migraine with aura [1], [2], [3]. SD is characterized as a near complete depolarization of neurons and glia, that slowly propagates through brain tissues at a rate of ∼2–5 mm/min. The event propagates due to the regenerative accumulation of extracellular K^+^ and/or glutamate at the wavefront, while the relative contributions of which depend on the initiating stimuli and the recording conditions [4], [5]. SD is a fully-recoverable event in healthy tissues, but can become deleterious when metabolic substrates are limited. We recently described the time course of excitatory transmission throughout SD, and reported that enhanced glutamate release and sustained NMDAR activation in the late phase prolongs the duration of depolarization and can trigger injurious Ca^2+^ load in metabolically compromised neurons [6]. Previous microdialysis studies have demonstrated significant elevation of extracellular GABA concentration under *in vivo* conditions of brain ischemia or K^+^ application where SD events are expected to occur [7], [8], [9], [10]. However the regulation and roles of GABA transmission at the SD wavefront, as well as contributions to excitability during the late SD phase remain to be elucidated.

Both *in vivo* and *in vitro*, the application of type-A GABA receptor (GABA_A_R) antagonists can be sufficient to generate SD, without other additional stimuli [11], [12]. GABA_A_R block also increased the propagation rate of events generated by localized K^+^ depolarization [13], suggesting that GABA_A_R activation is inhibitory for initiation and propagation [5]. Based on the effects described above, it might be expected that agents known to potentiate GABA_A_R function would decrease the incidence of SD. However a number of reports *in vivo* suggest that this is not the case [14], [15], [16], [17]. A recent study did show an inhibitory effect of a propofol precursor on SD incidence in mice, but it was suggested that this was not due to GABA_A_R effects of this agent [18]. Interestingly, a retrospective study of brain injury patients has suggested that propofol decreases incidence of SD [19] and, although SD was not monitored, several clinical studies have reported significant prevention of migraine attack by propofol [20], [21], which may be relevant if SD contributed to these events. It is currently unclear why GABA_A_R potentiators/agonists are not generally effective at inhibiting the initiation or propagation of SD, and whether or not propofol or related agents indeed potentiate GABA_A_R mediated currents to inhibit SD.

In the present study, we addressed the mechanistic basis for these apparently inconsistent effects of GABA modulators on SD by characterizing GABA_A_R mediated transmission throughout SD in acutely-prepared brain slices. A large GABA_A_R activation was detected during the very early phase of SD and both Cl^−^ measurements and the effects of antagonists implied that the current was inhibitory. Concentrations of propofol that effectively enhanced baseline GABA_A_R currents were without effect on events during SD, or SD propagation rates, likely due to a ceiling effect. We also found that GABA_A_R transmission was transiently depressed during the late phase of SD, a finding which may underlie the lack of effect of GABA_A_R modulators on the duration of depolarization in this vulnerable period.

## Methods

### Mouse and slice preparation

All experimental procedures were approved by the institutional animal care and use committee (IACUC) at the University of New Mexico. Brain slices were prepared from adult C57BL/6 mice (5–10 weeks old), by using our standard methods as described previously [6]. Brain slices (350 µm, coronal) were kept in ACSF (in mM: 126 NaCl, 3 KCl, 25 NaHCO_3_, 1.25 NaH_2_PO_4_, 2 CaCl_2_, 1 MgCl_2_, 0.4 ascorbate, equilibrated with 95% O_2_/5% CO_2_) at room temperature until used for experiments. All recordings were from hippocampal CA1 regions. Slices were set in a submerged recording chamber (RC-27, Warner Instruments, Hamden, CT) and superfused with ACSF at 2 ml/min and 32^°^C.

### Generation of spreading depolarization (SD)

SD was generated by microinjection of KCl into the slice (ejection volume ∼10 nl by a 40 psi, 10–40 ms pulse) by using a picospritzer (Parker Hannifin, Hollis, New Hampshire, USA), as described previously [6]. KCl pipettes site were placed >400 µm from recording sites. In all experiments, intrinsic optical signals (IOS: >575 nm light transmission through slices) were recorded as described previously to assess the propagation of SD [6]. SDs were also characterized by extracellular potential shifts and/or whole-cell recording (see below).

### Electrophysiology

All data were acquired by using a Multiclamp 700A amplifier, digitized with a Digidata 1334 and analysed by pClamp 9.2 (Molecular Devices, Sunnyvale, CA, USA). Extracellular potentials were acquired at 2 kHz, and all other electrophysiological data were acquired at 100 kHz. Extracellular potentials were recorded from the stratum radiatum using glass microelectrodes filled with ACSF (0.5–1 MΩ) inserted 100 µm below the slice surface.

Whole-cell clamp recordings were made from visually identified CA1 pyramidal neurons. Recordings were made by using a low chloride internal solution (in mM: 135 caesium metanesulfate, 8 NaCl, 10 EGTA, 5 QX314, 2 Na_2_ATP, 0.3 NaGTP, pH adjusted to 7.4 by CsOH). Neurons were initially clamped at −60 mV and intracellularly dialyzed at least for 10 minutes before SD challenge. Neurons with input resistance <150 MΩ at −60 mV were discarded. Holding potential was then increased to 0 mV. Recordings were made following inactivation of most voltage-gated currents. This was confirmed by monitoring the currents evoked by test voltage pulses (−10 mV, 100 ms test pulse), which gradually decayed over 5–10 minutes. Recordings of SD were initiated after obtaining stable baseline holding currents and input resistances. Due to the tissue swelling that occurs during SD, the recording electrode position was continuously adjusted throughout SD, as described previously [6]. Series resistance were tested after completion of each recording, and recordings were not analysed for neurons with access resistance larger than 25 MΩ or if >20% changes was detected following the recording.

Spontaneous inhibitory postsynaptic currents (sIPSCs) were detected and characterized by using Mini-analysis software (version 6.0.3, Synaptosoft, Decatur, GA), with a threshold amplitude of 7 pA. This detection parameter selectively detects GABA_A_R mediated responses. Because of the slow kinetics and short inter-event intervals, sIPSCs often overlapped and likely affected event detection. The potential contributions of these factors are discussed in the appropriate Results sections.

The amplitudes of GABA_A_R mediated tonic currents were calculated based on the offset of holding current generated by superfusion with gabazine (10 µM). Small oscillations (∼10 pA) of holding current were seen in some recordings, which made it difficult to obtain accurate assessments of tonic current amplitudes. Such recordings were excluded from the analysis.

### Extracellular Cl^−^ and TMA^+^ measurement

Extracellular chloride concentration ([Cl^−^]_o_) was measured by using chloride-selective glass microelectrodes. Theta-glass tubes were pulled to obtain ∼5 µm tips (5–10 MΩ in reference side). The reference barrel was filled with 150 mM NaCl and was also used to record the DC potential of SD. The ion-selective barrel was first silanized by sigmacote (Sigma Aldrich), and then back-filled with chloride ionophore cocktail A (Sigma Aldrich) and then backfilled with 150 mM NaCl. The ion-selective electrodes were inserted 100 µm below the slice surface. Data were acquired at 1 kHz with a 0.1 Hz low-pass filter. Calibrations were made by using standard solutions (15, 75 and 150 mM NaCl, Na^+^ concentration balanced to 150 mM with sodium gluconate) and [Cl^−^]_o_ concentration changes were then generated by using the Nikolsky equation as discussed in [22].

Extracellular volume estimations were made using TMA^+^ selective microelectrodes. The ion-selective barrel was made by tip-filling with an ionophore (IE190, WPA), followed by backfilling with 150 mM TMA-Cl. The reference barrel contained 150 mM NaCl. Recordings were made in the presence of 0.5 mM TMA-Cl in the bathing solutions. In the presence of TMA^+^ in pipette and bath solutions, the electrodes were highly selective for TMA^+^ and extracellular potassium generated negligible voltage responses (<1 mV) to [K^+^]_o_ increase from 3 to 30 mM. Electrodes were calibrated by using standard solutions (0.5, 1, 5 and 10 mM TMA-Cl, cation/anion concentration adjusted to 150 mM with NaCl).

### Statistics

All statistical analyses were made by using Graphpad Prism software (version 6.02, Prism, La Jolla, CA). Statistical tests were done by *t*-test or one-way or two-way ANOVA with post-hoc Turkey tests, unless otherwise described in Figure legends. Data are presented as mean ± SEM. In cases of repetitive experiments, paired data points are connected by lines. Throughout the manuscript, *n* values indicate the number of slices examined. A *p*-value <0.05 was considered statistically significant.

## Results

### Spontaneous IPSCs during SD

GABA_A_R mediated currents were isolated by holding at the reversal potential for glutamate receptor currents (0 mV) and by using low chloride intracellular solutions throughout the study (see Methods). Glutamate receptor antagonists were not used to isolate inhibitory currents, as these blockers significantly attenuate SD (Somjen, 2001). SDs were generated by KCl microinjection in hippocampus CA1 stratum radiatum, and detected by whole-cell clamp recording in individual CA1 pyramidal neurons, as well as extracellular DC recording in the adjacent dendritic subfield.

**Figure 1:**
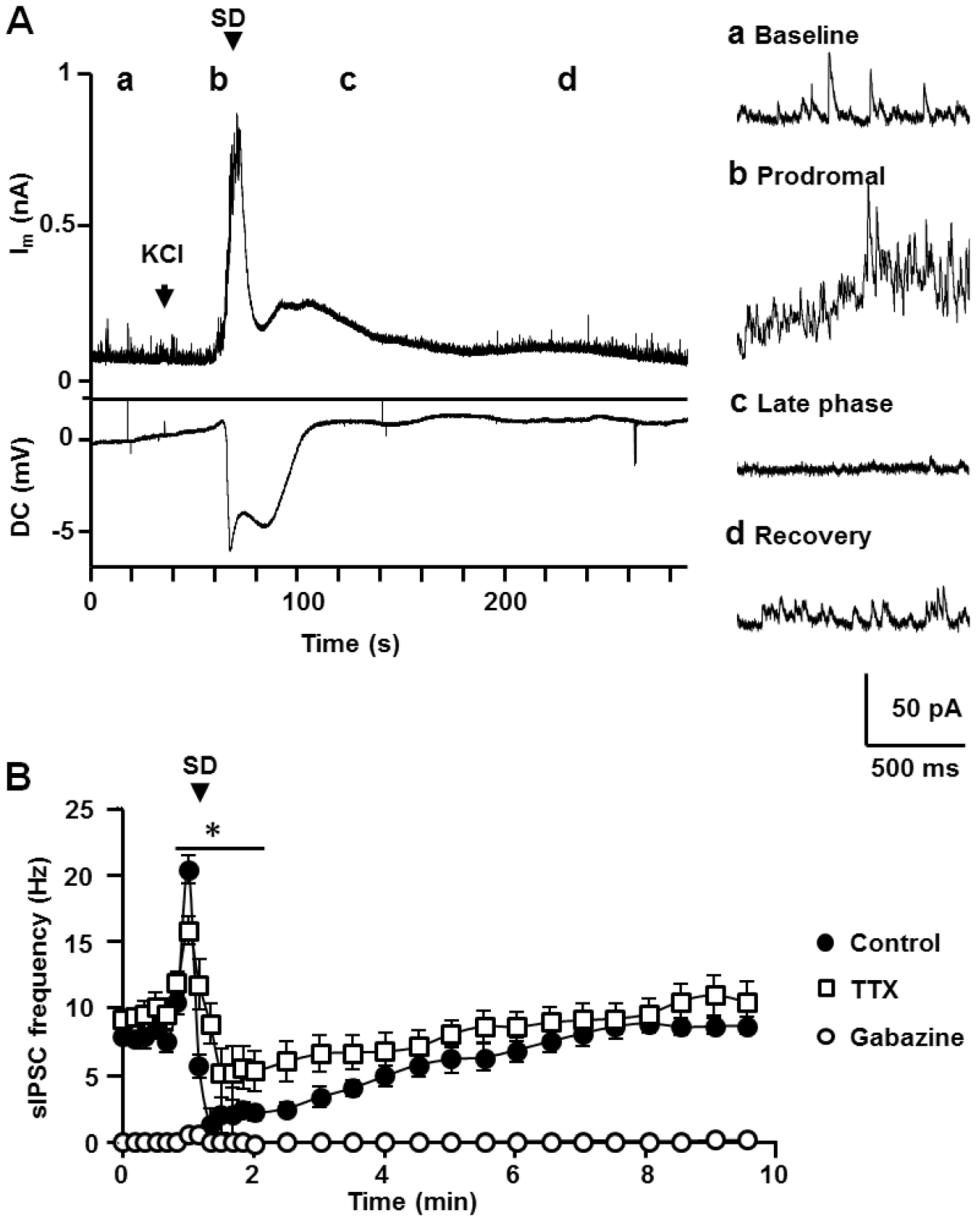
Characterization of sIPSC during SD. **A**. A representative recording of whole-cell current (I_m_, top) and DC potential (DC, bottom) during SD. Whole-cell recordings were made at 0 mV to isolate GABA_A_R mediated sIPSC. Right insets shows expanded spontaneous events during the periods indicated by **a-d**. The SD onset is defined by the onset of extracellular DC shift and is indicated by the arrow. There was a significant elevation of sIPSC frequency prior to SD arrival (**b**, Prodromal phase), followed by a transient suppression (**c**, late phase). **B**. Plots of mean sIPSC frequencies during SD are presented. Mean sIPSC frequency were calculated from 10 s bins during the initial 2 minutes, and later from 30 s bins. The arrow indicates the bin accompanying the onset of extracellular DC shift. Gabazine 10 µM (open squares) nearly completely eliminated sIPSC detection, and TTX 1 µM (open circle) decreased the initial sIPSC frequency increase as well as the degree of suppression. Note near complete elimination of sIPSC detection in the presence of gabazine (open squares). Statistical tests were performed between control and TTX. Control n = 9, Gabazine n = 6, TTX n = 7. *p<0.05, Control vs. TTX.

Figure 1A shows a representative recording of spontaneous inhibitory postsynaptic currents (sIPSCs) associated with SD. The frequency of sIPSCs increased during the 5–10 seconds prior to arrival of SD, termed here a “prodromal” phase (Figure 1Ab). A large outward current then began to develop 1–2 seconds prior to the extracellular DC potential shift that signified the arrival of SD. The peak of the large outward current usually coincided with the initial extracellular DC deflection, and then the current quickly decayed. The frequency of sIPSCs was greatly reduced immediately following the large outward current (1 minute window following peak SD current, termed here the “late phase of SD”, Figure 1Ac) and then recovered to baseline over the subsequent several minutes (Figure 1Ad).

Quantitative and pharmacological analyses of sIPSCs frequency associated with SD are presented in Figure 1B. The mean sIPSC frequency increase during the prodromal phase was significant (7.8±0.3 vs. 20.4±0.4 Hz, baseline vs. prodromal, p<0.001), as was the reduction in the late phase of SD (to 2.0±0.5 Hz, p<0.001, vs. baseline). The duration of the suppression period was quite variable (range 1.8–6.3 minutes, measured at 70% recovery), with an average duration of 4.3±0.4 minutes (n = 9). The GABA_A_R antagonist gabazine (10 µM), virtually abolished all sIPSCs recorded under these conditions during SD (n = 6), confirming that sIPSCs were due to GABA_A_R activation.

The mean sIPSC amplitudes during prodromal phase were significantly increased (15.1±0.6 vs. 32.4±2.7 pA, baseline vs. prodromal, p<0.001, n = 9) while the decay kinetics was not significantly altered (decay tau: 12.1±1.0 vs. 18.1±3.0 ms, p = 0.08, n = 9). During the late phase of SD, the mean sIPSC amplitudes decreased close to baseline levels (13.2±1.1 pA, p = 0.06 vs. baseline n = 9) and mean decay kinetics decreased to 9.2±0.6 ms (p<0.005 vs. baseline, n = 9). The larger mean sIPSC amplitudes during the prodromal phase could have increased detection of sIPSC. However by approximating the distribution of sIPSC amplitudes to the Poisson distribution [23], the numbers of subthreshold events (<7 pA) were estimated to be only 3.0±0.1% (range 1.4–6.2%) of the total events. Inclusion of such a small number of events could not explain the large mean sIPSC frequency increase during the prodromal (260% increase).

A potential influence of the decreased sIPSC amplitude during the late phase on sIPSC frequency was estimated using the same approach used above. When the mean amplitude was artificially decreased by 15% (difference in mean amplitudes from the baseline to the late-phase), subthreshold fraction was increased to 10.0±1.4% (range 5.0–16.0%) of total event. Again, such a small increase of subthreshold events cannot explain the decreased sIPSC frequency during the late phase (75% decreases).

Taken together, these observations imply that the changes in sIPSC frequency detected here were largely attributable to increased GABA release and/or receptor activation.

### Effects of TTX on sIPSCs during SD

The contribution of action potential-independent GABA release was examined by using tetrodotoxin (TTX, 1 µM, Figure 1B). TTX did not prevent SD (see discussion in [4]), and did not modify the amplitude of the large outward current recorded during SD (discussed below). As previously reported [24], TTX did not change baseline sIPSC frequency (9.6±1.0 Hz, p>0.1, vs. control, n = 7) or mean amplitude of events (14.1±0.9 pA, p>0.1, vs. control, n = 7). In contrast, during the prodromal phase, TTX significantly attenuated the elevation in sIPSC frequency (15.8±1.5 Hz, p<0.05, vs. control, n = 7) and prevented the increase in sIPSC amplitude (13.1±1.8 pA, vs. baseline, n = 7). TTX also attenuated sIPSC suppression in the SD late phase (mean frequency: 6.0±1.3 Hz, n = 7, p<0.05 vs. control).

### Relationship to excitatory currents during SD

**Figure 2:**
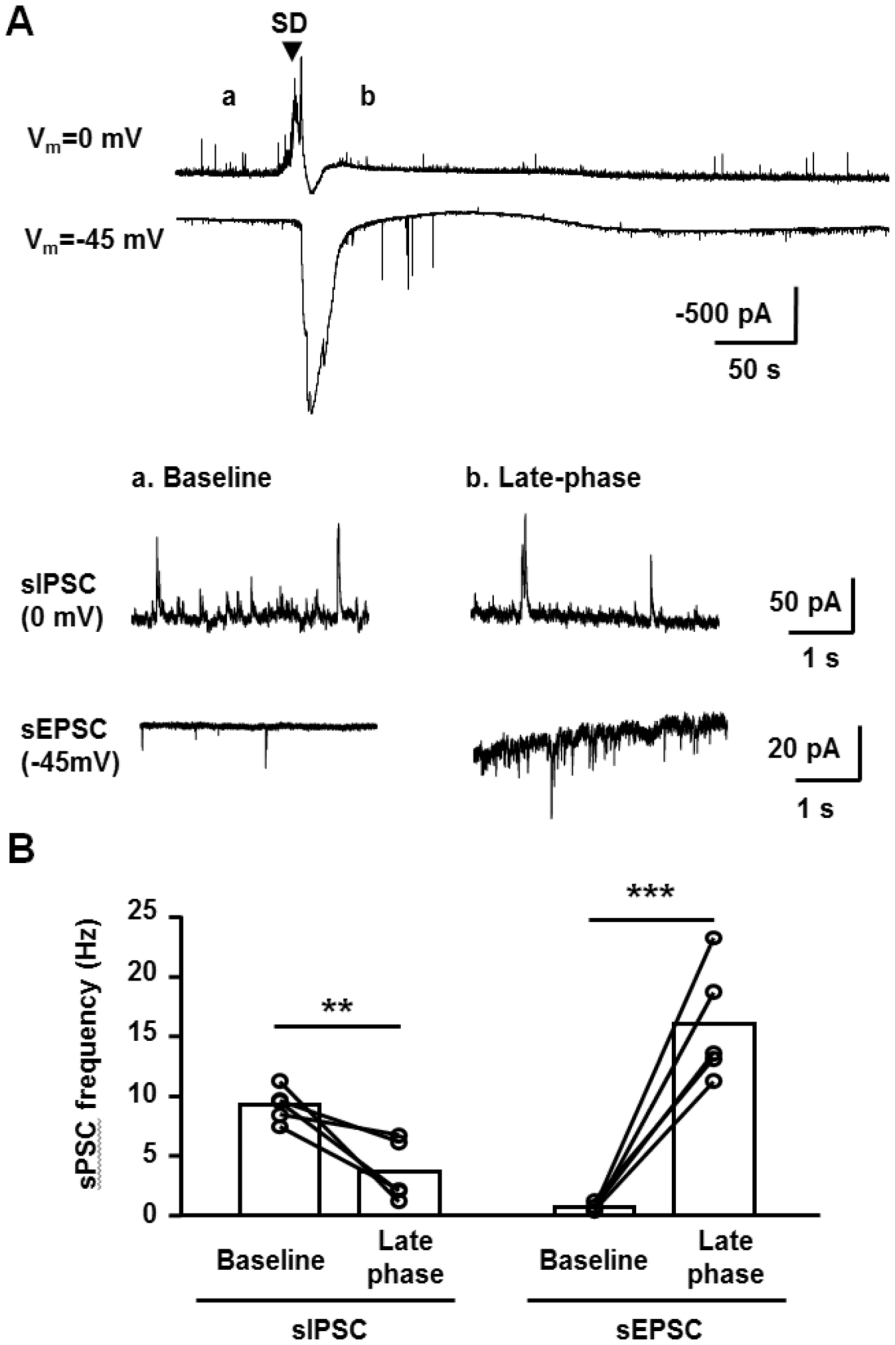
Comparison of sEPSC and sIPSC during SD. **A**. Top: representative recordings of pairs of SDs recorded in whole-cell configurations from the same neuron, either at 0 mV or −45 mV. SD onsets were aligned based on DC potential shifts and the onset is indicated by the arrow. Bottom insets show expanded sIPSC and sEPSC recordings during baseline (a) and the SD late phase (b) from the same recordings. **B**. Quantitative analyses from 5 sets of paired sIPSC and sEPSC recordings, showing frequencies during baseline and the late SD phase. In two cases, recordings from the same neuron could be maintained through two rounds of SD, and in the other three cases a newly-patched CA1 neuron was used for the second recording in the pair. **p<0.01, ***p<0.005, paired *t*-test, n = 5 each.

In contrast to the current observations with sIPSC suppression, we recently reported that sEPSC frequency *increased* during the late phase of SD [6]. This raised the possibility of a substantial increase of EPSC/IPSC ratio during the late SD phase. This was directly tested by making whole cell recordings during pairs of SDs at 0 mV and −45 mV in the same slices using the same intracellular recording solutions (**Figure 2**). Alignment of EPSC and IPSC recordings was performed based on the onset of extracellular potential shifts recorded with extracellular electrodes in each trial. These analyses confirmed significant opposing changes in sEPSC and sIPSC frequencies during the late SD phase (**Figure 2A&B**). Such an imbalance of IPSC/EPSC could underlie spiking activity and/or excitotoxic Ca^2+^ deregulation seen in the late SD phase (see Discussion).

Figure 2A also shows the earlier onset of the outward current relative to the large inward currents associated with SD (measured at 0 and −45 mV, respectively). This different time course was also demonstrated as an obvious biphasic current when detected at an intermediate holding potential (−10 mV, Figure S1). These responses further suggest that these currents are independent anion selective and non-selective cation conductances, respectively. This is consistent with the fact that the early currents were eliminated by GABA_A_R antagonist (see below). The time course of the outward and inward currents also imply that the rapid decay of outward current was at least in part due to loss of membrane resistance associated with the large inward SD current. Other mechanisms such as GABA depletion and reduced driving force (i.e. as a consequence of [Cl^−^]_o_ decrease, see below) may also contribute to the decay of the outward current (see Discussion).

### Potential mechanisms underlying sIPSC suppression

We next explored potential mechanisms underlying sIPSC suppression during the late phase of SD. Postsynaptic Ca^2+^ elevations are profound during SD [6], [25], [26], [27] which could have decreased postsynaptic GABA_A_R currents [28], [29], [30]. Thus we tested the effects of intracellular dialysis with the fast acting Ca^2+^ chelator 1,2-bis(o-aminophenoxy)ethane- N,N,N',N'-tetraacetic acid (BAPTA, 10 mM, replacing EGTA in the pipette solution). BAPTA significantly increased the baseline sIPSC frequency (13.7±1.0 Hz, n = 4, p<0.005), confirming that sIPSC frequency was sensitive to the [Ca^2+−^]_i_ in these preparations. However BAPTA did not prevent sIPSC suppression following SD (late-phase frequency: 3.0±1.0 Hz, 70% recovery: 3.6±0.8 minutes, n = 4, compare with Figure 1B). Depression of GABA release by presynaptic cannabinoid receptor (CB_1_R) activation [31] was also tested. Suppression of sIPSCs was still observed in the presence of the CB_1_R antagonist SR141716A (10 µM) (late-phase frequency: 3.1±1.5 Hz, 70% recovery: 3.1±0.5 minutes, n = 4). These results suggest that mechanism(s) other than postsynaptic mechanism contributed to the IPSC suppression (see Discussion).

### Characterization of evoked IPSCs

**Figure 3:**
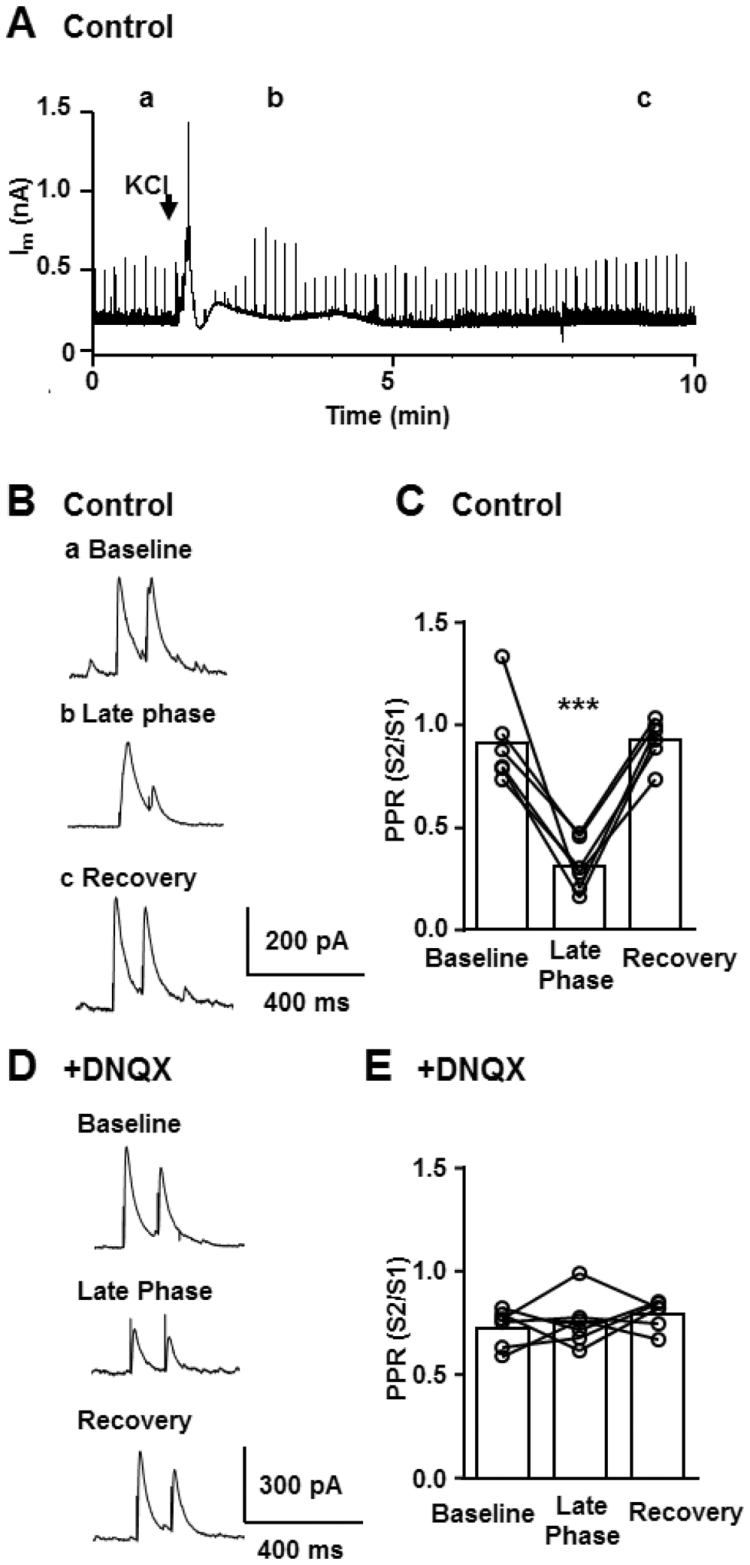
Characterization of paired pulse ratio (PPR) of evoked IPSC during SD. **A-C**. Characterization of PPR in control conditions. Pairs of IPSC (100 ms pulse interval, 0.1 Hz) were continuously evoked throughout whole-cell recordings of SD, and the PPR in different phases of SD were analysed. In the representative trace (**A**) evoked IPSCs are represented as sharp vertical lines. **B**. Single evoked IPSC pairs at corresponding time points are presented at an expanded time scale (**a-c**). Note the change in paired-pulse ratio as well as slow kinetics of IPSC during late phase (**b**). **C**. Quantitative analysis of PPR during control, SD late phase and following full recovery. ***p<0.005, n = 6. **D&E** The same experiments were conducted in the presence of the AMPA receptor antagonist DNQX (20 µM) to isolate monosynaptic components. **D**. Representative IPSCs are shown. Note that DNQX nearly completely abolished PPR changes during the late phase. **E**. Quantitative analysis of PPR changes. There was no significant change in PPR of monosynaptic IPSCs. n = 6.

We next evaluated whether decreased initial GABA release probability may contribute to decreased sIPSC frequency in the late phase. This was done by analyzing the paired pulse ratio (PPR) of evoked IPSC (eIPSC) which can provide an indirect assessment of presynaptic GABA release probability (**Figure 3**). Pairs of eIPSCs (100 ms pulse interval) were evoked by using a bipolar electrode (Methods). Bipolar stimulation often failed to evoke IPSCs during the prodromal phase, and Figure 3A shows that eIPSCs were suppressed during the SD late phase. It is noted that eIPSCs could always be detected earlier following SD (70% recovery of amplitude: 1.5±0.5 minutes), compared with the recovery of sIPCS frequency in the same preparations (70% recovery: 3.9±0.4 minutes, n = 6).

In control conditions, baseline PPR was stable within individual recordings. In the late SD period, PPR was expected to increase, as would be expected from sIPSC suppression due to decreased initial release probability during this phase. On the contrary, PPR was strongly depressed (**Figure 3B&C**), suggesting an increase in initial release probability. However it was also noted that the kinetics of evoked IPSC were significantly prolonged during the late phase (decay tau: baseline: 20.1±2.0 ms, late phase: 48.9±7.5 ms, n = 6, p<0.05), which raised the possibility that the increased detection of polysynaptic eIPSCs hindered the detection of initial release probability based on PPR [32]. Thus similar experiments were conducted in the presence of the AMPA receptor antagonist DNQX (20 µM) to isolate monosynaptic eIPSCs [33]. SD was still reliably generated, and DNQX prevented changes in both PPR (**Figure 3D&E**) and eIPSC kinetics in the late phase (decay tau: baseline: 18.7±1.8 ms, late phase: 20.6±2.2 ms) consistent with the idea of increased polysynaptic components during the SD late phase. Thus despite the significant suppression of sIPSC, there appears to be an increase in initial GABA release probability, due to polysynaptic response. Such increased release probability however did not contribute to the recovery rates of eIPSC or sIPSC as DNQX did not affect these parameters (70% recovery times; eIPSC amplitude: 1.3±0.3 minutes, sIPSC frequency: 3.4±0.8 minutes, both p >0.05, vs. control, n = 6). Together, these results suggest that sIPSC suppression following SD is unlikely explained by decreased GABA release probability alone, and may involve other mechanism such as vesicular GABA depletion associated with a large metabolic burden of SD (see Discussion).

### Characterization of the large GABA_A_R current during the early phase of SD

**Figure 4:**
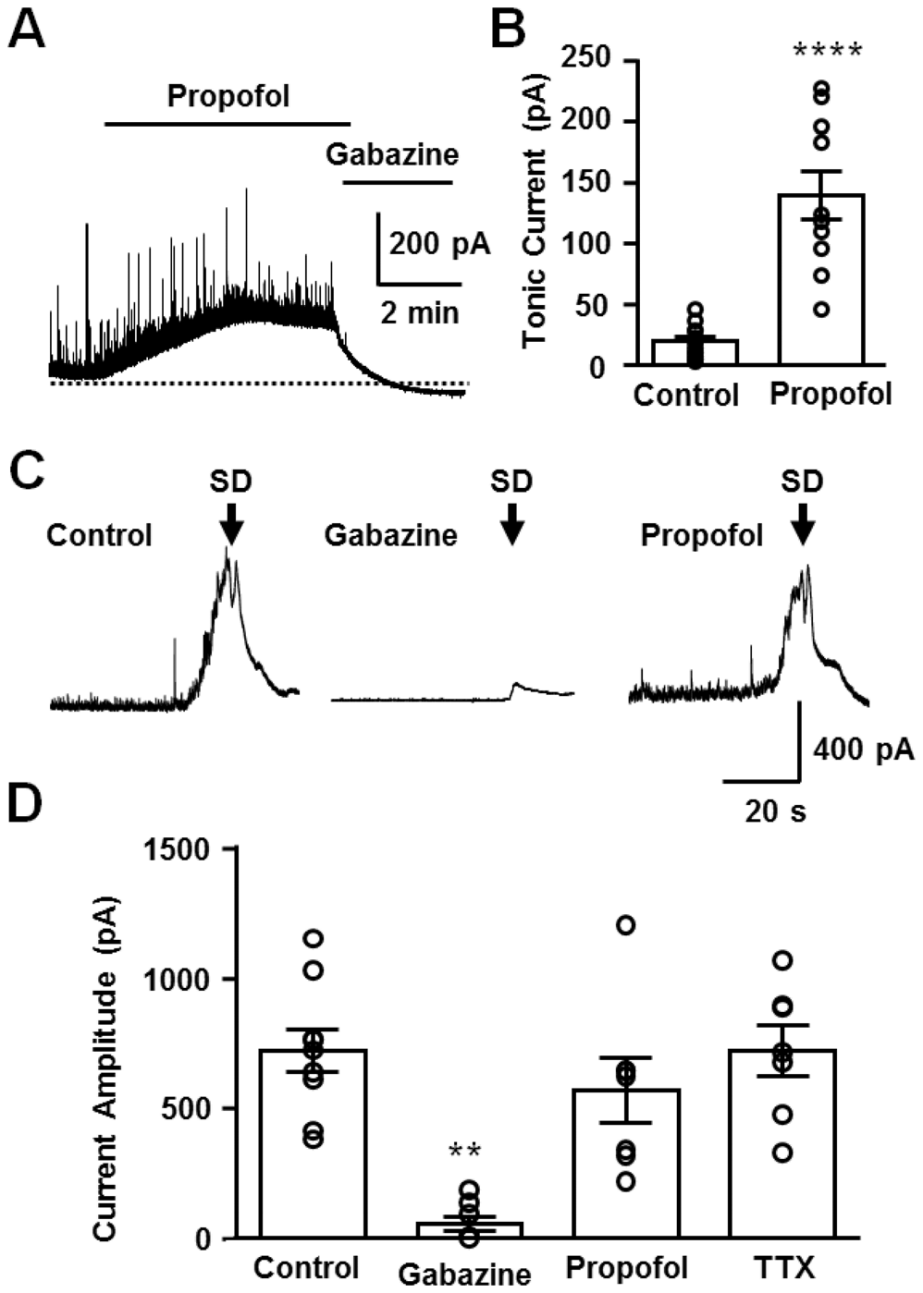
Effects of propofol and gabazine on the large GABA_A_R current during SD. **A&B** Effects of propofol (200 µM) and gabazine (10 µM) on basal GABA transmission. Representative traces (**A**) and quantitative analysis (**B**) are shown. ****p<0.001, Control: n = 15, propofol n = 10 **C&D** Pharmacological characterization of the large GABA_A_R current during SD. Gabazine nearly completely eliminated the early GABA_A_R current, while propofol had little effect. Representative traces of early GABA_A_R currents in control conditions as well as in the presences of gabazine and propofol, at the concentrations used in **A&B**. The SD onsets are defined by the onsets of extracellular DC shifts and are indicated by the arrows. **D**. Quantitative analysis of current amplitudes. Current amplitudes were determined from holding currents immediately before SD onset. Control: n = 9, gabazine: n = 8, propofol: n = 7, TTX: n = 7, **p<0.01 compared to others.

The voltage-sensitivity of the large outward current shown in Figure 1A is consistent with a GABA_A_R-mediated event, and **Figure 4** shows that the GABA_A_R antagonist gabazine (10 µM) nearly completely eliminated the large outward current seen during the early phase of SD (Figs. 4C&D). We then examined the effects of the GABA_A_R potentiator/agonist propofol. A series of pilot studies showed that propofol did not interfere with the initiation or propagation of SD across a range of test concentrations (15–500 µM). For subsequent studies, a near-maximally effective concentration was used (200 µM), which elevated tonic GABA_A_R currents by ∼7 fold (Figs. 4A&B). Despite this large potentiation of baseline GABA_A_R current, propofol did not affect the large GABA_A_R current recorded during SD (Figs. 4C&D). This could suggest a near maximum activation of these channels by endogenous GABA during SD (see Discussion).

**Figure 5:**
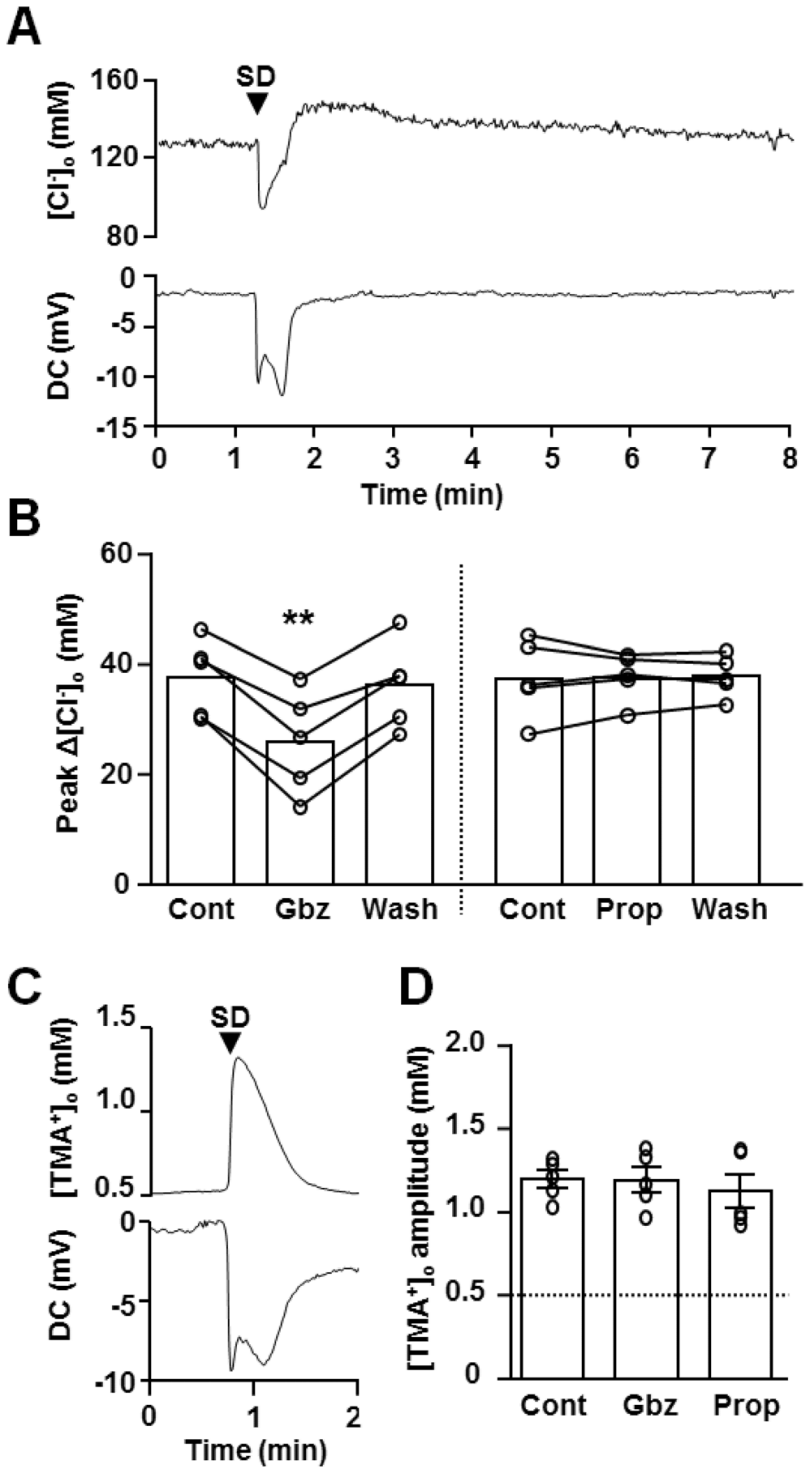
GABA_A_R activation during SD contributes to cellular Cl^−^ influx. **A&B** [Cl^−^]_o_ during SD were measured by using Cl^−^ selective electrodes. **A**. Representative [Cl^−^]_o_ change during SD, characterized as an Initial decrease and a subsequent slow overshoot response. Extracellular DC potential recorded from the reference barrel is also shown below. **B**. Pharmacology of [Cl^−^]_o_ was tested in repetitive SD. Gabazine (Gbz, 10 µM) significantly decreased [Cl^−^]_o_ decreases during SD, while propofol (Prop, 200 µM) was without effect. **p<0.01 **C&D** Extracellular volume changes were analysed by assessing bath applied TMA^+^ (0.5 mM) concentration changes. [TMA^+^]_o_ in slice were measured with TMA^+^ selective electrodes. Representative plots of [TMA^+^]_o_ and DC potential shift are shown in **C** and quantitative analysis of peak amplitudes are shown in **D**. Gabazine and propofol had no significant effect on [TMA^+^]_o_ responses.

We next examined whether GABA_A_R activation generates outward current (i.e. Cl^−^ influx) or alternatively whether the initial large GABA_A_R activation rapidly elevates [Cl^−^]_i_ to levels sufficient to produce a depolarizing GABA_A_R current. This was evaluated indirectly by measuring extracellular Cl^−^ ([Cl^−^]_o_) concentration changes during SD (**Figure 5A**). Chloride sensitive electrodes were set in the CA1 dendritic subfield and showed that [Cl^−^]_o_ rapidly dropped to 88.3±3.2 mM (n = 5) when the deflection of extracellular DC shift first appeared. [Cl^−^]_o_ always recovered when extracellular potential shifts began to decay, and later generated a transient overshoot. In most experiments, the [Cl^−^]_o_ overshoot recovered within a few minutes to baseline levels. These biphasic [Cl^−^]_o_ responses during SD were previously reported *in vivo* rat cortex and overshoots was suggested to be due to Cl^−^ pumping activity [34]. Reduction in Cl^−^ influx due to the sIPSC depression and effects of shrinkage of extracellular space during SD (see below) could also contribute to [Cl^−^]_o_ overshoots.

Pharmacological analyses of [Cl^−^]_o_ changes were conducted in SDs generated repetitively in same slices. Gabazine significantly reduced the amplitude of [Cl^−^]_o_ decreases (66.8±6.3% of control, n = 5, **Figure 5B**), and this gabazine effect was reversed after wash out (95.6±2.6% of control, n = 5). On the other hand, gabazine had no significant effect on the [Cl^−^]_o_ overshoot (141.8±3.5 vs. 141.2±2.2 mM, control vs. gabazine n = 5, p >0.1). Propofol was without effect on the amplitude (**Figure 5B**) and overshoot of [Cl^−^]_o_ change during SD (143.0±1.7 vs. 143.6.2±0.7 mM, control vs. propofol, n = 5, p >0.1).

During SD, significant tissue swelling occurs and the degree of extracellular space shrinkage could affect [Cl^−^]_o_ measurements. We thus evaluated extracellular space changes during SD by measuring bath-applied TMA^+^, using an approach described previously [35]. TMA^+^ is relatively cell-impermeable and TMA^+^ concentration increases indirectly report extracellular space shrinkage. TMA^+^ itself had little effect on SD propagation rate or DC shifts. However prolonged exposure occasionally prevented recovery from SD. Thus TMA^+^ measurements were conducted on single SDs generated in each slice, rather than repetitive SD. Upon SD arrival, [TMA^+^]_o_ concentration increased in a monophasic manner (**Figure 5C**), and the kinetics of the rising phase was comparable with that of [Cl^−^]_o_ decreases. Both gabazine and propofol did not significantly modulate peak [TMA^+^]_o_ accumulation (**Figure 5D**), implying that tissue swelling differences do not account for the attenuated [Cl^−^]_o_ decreases by gabazine (see Figure 5B).

The significant decrease in extracellular space implied a profound increase in the [Cl^−^]_i_ more than expected from [Cl^−^]_o_ measurement during SD (see Discussion). Nonetheless, our results collectively suggest that GABA_A_R activation during SD has a hyperpolarizing influence (i.e. Cl^−^ influx) during the SD early phase, which should counteract neuronal depolarization.

### Effect of GABA receptor modulators on the characteristics of SD

**Figure 6:**
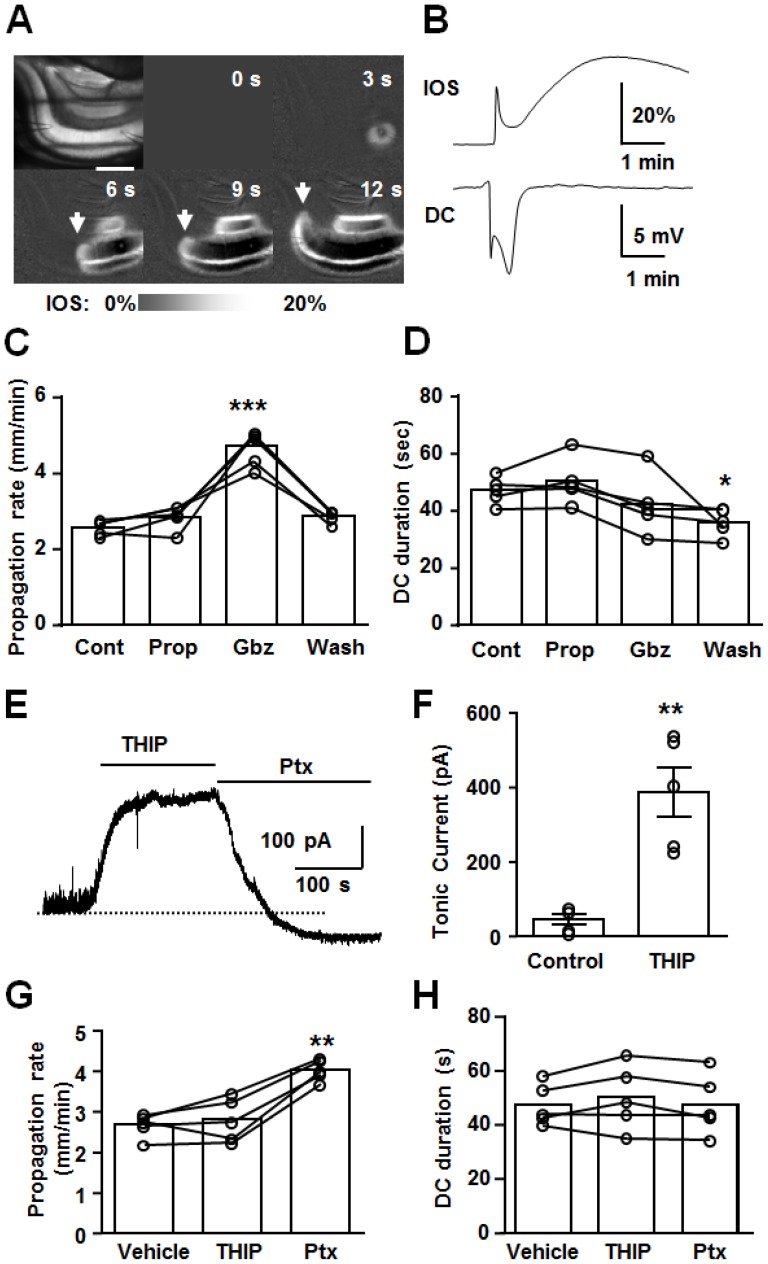
Effects of GABA_A_R potentiation and inhibition on the propagation rates and durations of SD. **A&B**. Representative recording of SD generated in hippocampal CA1. **A**. Image sequence shows IOS signal during SD. Left top panel shows a raw image and others are ratio images of IOS. Scale bar: 400 µm. A KCl microinjection resulted in increased light transparency as indicated by increased IOS traveling across CA1 subregion (left panels, arrow indicate wavefront). SD arrival was indicated by a biphasic DC potential shift and enhanced IOS signal as indicated in **B**. **C&D** Effects of propofol (Prop, 200 µM) and gabazine (Gbz, 10 µM) on SD propagation rate and duration of DC shift (70% recovery) were evaluated by repetitively generated SD. In these experiments, 4 SDs were generated in single slices with different conditions (i.e. control, propofol, gabazine and wash, 10 minutes drug exposures or 20 minutes washout,>15 minutes recovery after SD). n = 5, ***p<0.005, * p<0.05. **E&F** Effects of THIP (10 µM) and picrotoxin (Ptx, 50 µM) on sIPSC and tonic current. n = 5, ** p<0.01. **G&H** Effects of THIP and Ptx on the SD propagation rate and DC duration. n = 5, **p<0.01.

The results above suggest an inhibitory role for GABA_A_-R activation during SD, which is most prominent during the onset of SD and is suppressed during the SD late phase. We next tested whether the effects of GABA_A_R modulators described above were matched by effects on SD propagation rates and the duration of DC shifts (**Figure 6**). Propagation rates were characterized from imaging of intrinsic optical signals (**Figure 6A&B**), and 4 SDs were generated each in single slices, with sequential exposures to test agents (e.g. control, propofol, gabazine and wash, Figs 6C&D). Propofol was without effect on both SD propagation rate and the duration of DC potential shift. Gabazine significantly enhanced propagation rates, but was without effect on durations of DC potential shift. After gabazine washout, SD propagation rates recovered to control levels, but DC shift durations were always shorter that control values (see [36]). Similar effects were observed with another agonist/antagonist pair, 4, 5, 6, 7-tetrahydroisoxazolo[5, 4-c]pyridin-3-ol (THIP, 10 µM), and picrotoxin (Ptx, 50 µM). In control experiments, we confirmed that bath application of THIP significantly increased tonic currents while Ptx abolished sIPSC and the tonic current (**Figure 6E&F**). GABA_A_R activation by THIP was without effect on SD propagation while the GABA_A_R antagonist Ptx accelerated it (**Figure 6G**). These results are consistent with strong GABA_A_R activation during the early SD phase, that limits the propagation rate of the SD wavefront. Durations of DC potential shifts were unaffected by these drugs (**Figure 6H**). The absence of effect on the duration of DC potential shift could be explained by the absence of endogenous GABA_A_R activation during the SD late phase, in addition to other mechanisms (see Discussion).

Finally, we examined effects of the GABA_B_Rs agonist baclofen and antagonist SCH-50911. In a set of pilot experiments, we confirmed effective concentrations of these agents by evaluating effects on evoked field EPSPs. Baclofen (10 µM) effectively suppressed evoked field EPSP (15.3±0.5% baseline, n = 5) and the inhibition could be reversed by further addition of 10 µM SCH-50911 (103.2±1.1% baseline, n = 5). We then tested these concentrations on SD characteristics, using the same experimental design as shown in Figure 6. In contrast to GABA_A_R effects, the agonist baclofen significantly decreased SD propagation rates (2.68±0.1 vs. 1.9±0.1 mm/min, control vs. baclofen, p<0.05, n = 5), while the antagonist SCH-50911 did not show any significant effect on propagation (2.92±0.1 mm/min, n = 5). Both agents were without effect on the duration of the DC potential shifts (45.5±2.3, 39.4±3.1, 46.7±2.1 s; control, baclofen, SCH-50911 respectively; p >0.05, n = 5). These results suggest that exogenous GABA_B_Rs activation can inhibit SD propagation, but the lack of effect of the antagonist implies that activation of GABA_B_R by endogenous GABA does not make a significant contribution to SD propagation under these conditions.

## Discussion

### Summary

This study characterized GABA_A_R activation during SD, and investigated the basis for effects of GABA_A_R modulators. Whole-cell recordings revealed a large GABA_A_R activation, primarily during the early phase of SD, which generates inhibitory outward current (Cl^−^ influx) to limit SD propagation. The endogenous activation of GABA_A_Rs during SD appears strong enough to prevent additional effects of pharmacological potentiation. During the late phase of SD, spontaneous GABA transmission was suppressed, which resulted in an imbalance between excitatory and inhibitory inputs during this phase. These findings provide insight into mechanisms underlying the effects of the GABA_A_R modulators on SD, and help explain some controversial effects of GABA_A_R modulators on SD under different experimental or clinical conditions.

### Inhibitory GABA_A_R activation during the early SD phase

Significant GABA release during SD and related events have been reported in previous *in vivo* studies (see Introduction). The present study implies that GABA release occurred during the early phase of SD and mostly activates GABA_A_Rs, to generate outward current (Cl^−^ influx). Inhibitory GABA_A_R currents at the advancing SD wavefront would be expected to contribute to effects of GABA_A_R antagonists on propagation.

It was noted that the onset of outward GABA currents preceded inward depolarizing currents at the onset of SD (Figure 2). This may seem surprising, if there is simultaneous triggering of excitatory and inhibitory inputs at the SD wavefront, and the mechanism(s) underlying this observation are not known. It is possible that the large inward current is relatively delayed because of a reliance on voltage-dependent conductances. In addition, it is possible that the higher frequency and the slower decay kinetics of GABA_A_R currents allow individual GABA_A_R IPSC (rather than AMPAR mediated EPSC, Figure 2) to combine more readily form a large current during the early SD phase, when release probability is increased (see Figure 1).

In contrast to the present study, GABA_A_R antagonism inhibited SD generation in preparations from immature animals [36], where GABA_A_Rs are expected to generate depolarizing current. Depolarizing GABA_A_R currents could also arise in adult preparations as a consequence of down-regulation of KCC2 by ischemia [37] and/or overactivation of NMDARs [38]. Under such conditions, the early GABA_A_R activation during SD may significantly facilitate SD propagation.

In contrast to the clear effects of antagonists, GABA_A_R potentiation by propofol had little effect on the large GABA_A_R current during SD and was without effect on SD propagation. The propofol concentrations tested here were up to ∼20-times higher than those used clinically [39] and showed strong potentiation of basal GABA_A_R currents (Figure 4), validating effectiveness of the drug under these conditions. The ineffectiveness of propofol on SD may instead be due to ceiling effects of GABA_A_R currents, during the strong endogenous receptor activation during the early phase of SD. It has been shown that propofol is not able to increase GABA_A_R activation when ambient GABA concentration is 30–100 µM [40], [41]. The extracellular concentrations of GABA achieved during the peak of SD are not easy to estimate, since microdialysis studies usually require integration of signals over time spans that are significantly longer than the passage of the SD wave-front. The ability to potentiate GABA_A_R mediated effects may also be limited by the reduced driving force for Cl^−^ influx during the peak of SD, similar to previous descriptions of GABA_A_R current “fade” [42], [43].

The lack of effects of propofol on GABA_A_R currents supports the notion that GABA_A_R effects do not account for inhibitory effects of propofol or its precursor on SD in uninjured brain ([18], see Introduction). Positive effects of propofol that have been suggested from previous clinical studies of acute head injury and migraine (see Introduction) may be due to other effects of this agent (e.g. antioxidant, metabolic and/or cerebral perfusion) [39], [44], [45]. Interestingly, the recent retrospective study of SD in brain injured patients that showed decreased clusters of SDs by propofol also reported that an alternative GABA_A_R activator (the benzodiazepine midazolam) actually increased SD incidence [19], further suggesting that GABA_A_R activation may not be a useful pharmacological approach for SD suppression.

### Extracellular chloride dynamics during SD

The peak amplitude and kinetics of [Cl^−^]_o_ dynamics recorded here during SD were similar to those recorded in rodent cortex *in vivo* [34], [46]. The biphasic nature of the [Cl^−^]_o_ response was previously attributed to Cl^−^ pumping activities [34]. In the current study, comparison with IOS responses implies that significant tissue swelling persists during the [Cl^−^]_o_ overshoot (see Figure 5A and Figure 6B) and it is thus possible that decreases in the extracellular space during the SD late phase contributed to the overshooting [Cl^−^]_o_ recovery. We note that [TMA^+^]_o_ measurements did not detect the slow swelling response (see Figure 5C vs. Figure 6B), likely due to diffusion and eventual equilibration of the elevated [TMA^+^]_o_ to the external solution, even when the extracellular space remains constricted (see also [47]).

The present study revealed that the overshooting recovery of [Cl^−^]_o_ coincided with sIPSC suppression. We therefore suggest that sustained decreases in the extracellular space, concomitant with reduced Cl^−^ flux through GABA_A_R during the SD late phase could together be significant mechanisms of the [Cl^−^]_o_ overshoot.

The assessment of initial extracellular volume by TMA^+^ measurements showed ∼250% decrease in the extracellular volume (see [35]), and implies that [Cl^−^]_i_ elevates much more than expected from [Cl^−^]_o_ measurement alone. It seems feasible that very large neuronal [Cl^−^]_i_ increases through other channels could limit GABA_A_R-mediated inhibitory effect on SD. Direct measurement of [Cl^−^]_i_ during simulated ischemia was previously demonstrated by bulk-loading of fluorescence Cl^−^ probe 6-methoxy-N-ethlquinolinium (MEQ) [37]. This MEQ indicator approach was also tested in the current study, but was found to be unreliable when tested with single-neuron loading, because of substantial neuronal indicator loss during SD and a large contamination of tissue autofluorescence changes.

Our results indicate that GABA_A_R activation contribute to only ∼30% of [Cl^−^]_o_ decrease during SD, and thus the majority of [Cl^−^]_o_ decreased during SD is mediated by other mechanisms. Because the outward current during SD was entirely due to GABA_A_R in pyramidal neurons (Figure 4), non-electrogenic Cl^−^ transporters in neurons and/or Cl^−^ conductances present in other cell types [48] likely contributed to the bulk of [Cl^−^]_o_ decreases during SD. Cl^−^ fluxes have previously been suggested to be involved in the initiation, but not progression of SD generated by hypoxia, and identification of these pathways may allow indirect modulation of inhibitory GABA_A_R activation during SD.

### Synaptic transmission in the SD late phase

The suppression of sIPSC during the SD late phase is in sharp contrast to the enhanced glutamate transmission during the same period (Figure 2, see also [6]). The differential effects on sEPSC and sIPSC frequencies during this phase may underlie the observation that the duration of DC shifts was reduced by NMDAR antagonists [6], but unaffected by GABA_A_R antagonists (Figure 6D&H). The mechanisms contributing to the increased EPSC/IPSC ratio are not yet known, but could include differential regulation of glutamate vs. GABA synthetic pathways under the metabolic stress of SD. It was previously suggested that GABA synthesis is more vulnerable to substrate depletion (hypoglycemia [49]), and it is possible that a similar depletion could occur after the profound metabolic challenge associated with SD. GABA_A_R agonist/potentiators did not modulate the duration of DC shifts. A previous study suggested a depolarization shift of the reversal potential for GABA_A_R current during recovery from SD generated by anoxia [50], likely due to elevation of [Cl^−^]_i_. It is possible that, in the current conditions, the electrochemical gradient of GABA_A_R was close to neutral during the late SD phase, and exogenous GABA_A_R agonists (propofol and THIP) could not generate charge transfer sufficient to modulate cellular depolarization.

The late phase of SD may be particularly important for determining recovery of neurons in metabolically compromised conditions, as sustained NMDAR activation during this phase could lead to Ca^2+^ overload and membrane compromise [6]. Interestingly, a GABA_A_R antagonist bicuculline has been shown to generate spiking activity during the late SD phase in epileptic brain tissues [51] and in normal tissues [52]. It is possible that GABA signalling and Cl^−^ homeostasis are altered under such conditions, and modulated excitability of this vulnerable period.

The present study suggested that sIPSC suppression during the late phase was not contributed to by Ca^2+^ dependent GABA_A_R down-regulation, or cannabinoid receptor-1 (CB_1_R) mediated depolarization induced depression of presynaptic GABA release (see Results). The presence of evoked IPSC during the suppression period suggests that the sIPSC suppression is unlikely due to intracellular Cl^−^ accumulation or compromised quality of voltage-clamp. It is possible that depletion of GABA due to the large GABA release early in SD events [53], [54] and/or decreased GABA synthesis by altered energy metabolism following SD [49] may contribute to this suppression. However it was technically challenging to analyse vesicular content, since the additional mechanical stress of pressure pulses required for application of hyperosmolar solutions applications, compromised our whole-cell recordings, when combined with the movement already observed during SD.

In the late SD phase, evoked IPSC became detectable much earlier than the recovery of sIPSC frequency. While these recovery rates cannot be directly compared, the different recovery rates may be partly due to the contribution of different interneuron populations to spontaneous and evoked responses. The enhancement of evoked IPSC seen in our whole cell recording extends previous reported short-term alteration of evoked IPSP during hypoxic SDs [50]. In addition to the short-term alteration shown here, previous *in vivo* studies have also suggested much longer lasting (hours) modulation of inhibitory signalling after SD, based on either paired-pulse ratio changes [55] or changes in compound extracellular evoked potentials [56]. Such long-term modulations of evoked IPSC were not observed here, and in fact evoked IPSC responses returned to baseline level within a few minutes. This raises possibilities that absence of eIPSC suppression was tissue specific, or previously described effects were not mediated by GABA.

### Conclusions

The present study revealed a large inhibitory GABA_A_R activation during the progression of SD, which likely acts as a limiter of propagating event. Strong endogenous activation of GABA_A_Rs during SD appears to underlie the ineffectiveness of potentiation of these currents to limit SD events, and approaches to maintain the driving force for inhibitory Cl^−^ flux may be a useful approach to improve neuronal repolarization.

## Supporting Information

Figure S1 **Whole-cell current recorded from a CA1 pyramidal neuron during SD.** The recording is as described for Figure 1, with the exception that the holding potential here was −10 mV, rather than 0 mV. At this holding potential, the outward and inward phases of the currents during SD are more clearly distinguished; the early outward current being followed by a clear inward current.
(TIF)
